# Supplementary material for: Carbonic Anhydrase VIII (CAVIII) Gene Mediated Colorectal Cancer Growth and Angiogenesis through Mediated miRNA 16-5p
Source: Biomedicines. 2022 Apr 29;10(5):1030. doi: 10.3390/biomedicines10051030 (PMC9138292; doi:10.3390/biomedicines10051030)
Supplement: Supplementary file 1 [file biomedicines-10-01030-s001.zip › biomedicines-1691112-supplementary.pdf]

Supplemental Data

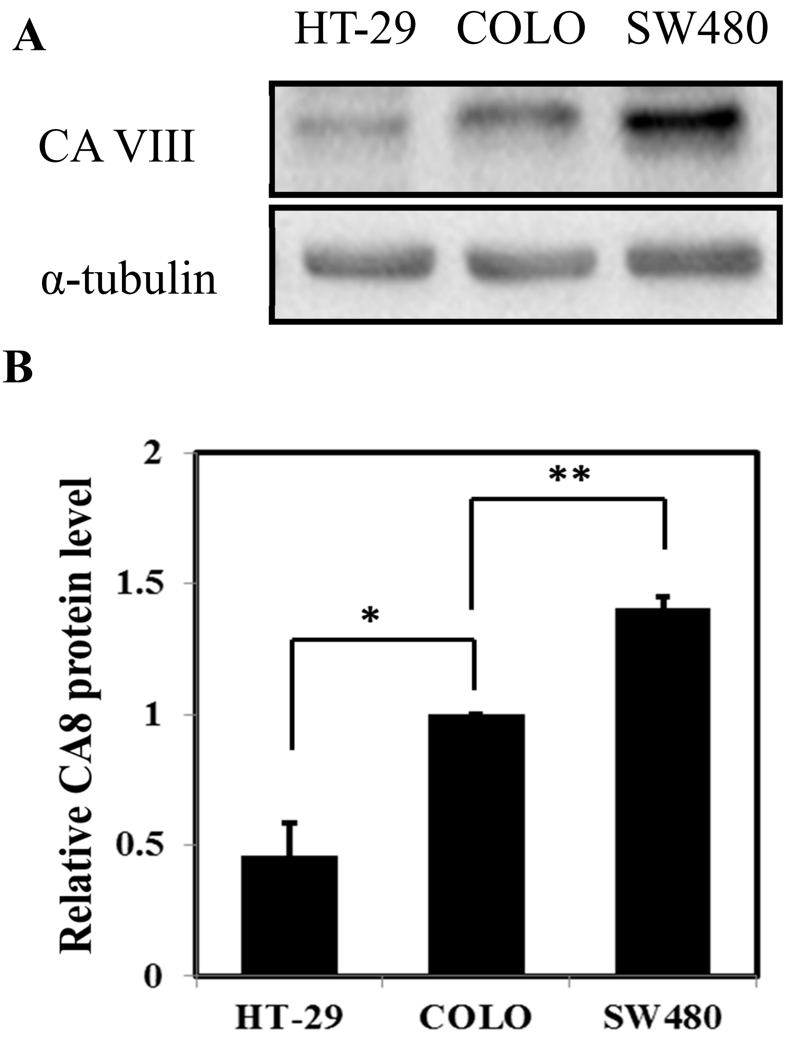

Figure S1. Western blot analysis of CA8 expression in different colon cancer cell lines. (A) Various endogenous CA8 expressed in a panel of colon cancer cell lines including HT29, COLO320, and SW480. Expression of CA8 was examined by immunoblotting whole cell lysates from different colon cancer cell lines. (B) Expression of CA8 in HT29, COLO320, and SW480 with Quantitative Data.

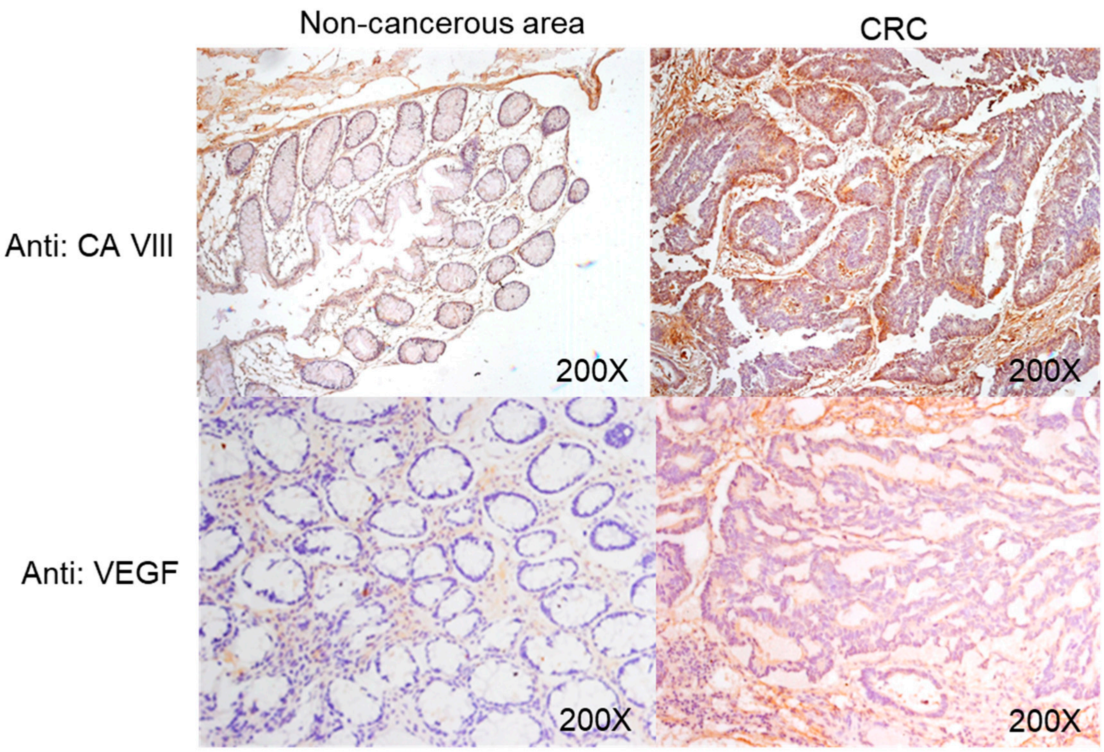

Figure S2. Expression of PC in paraffin-embedded tissues of CRC patients by IHC staining (B) and (D). (A) and (C) indicate the non-cancerous area adjacent to the cancerous area in CRC, respectively. Original magnification 200×. Scale bar; 100 µm.

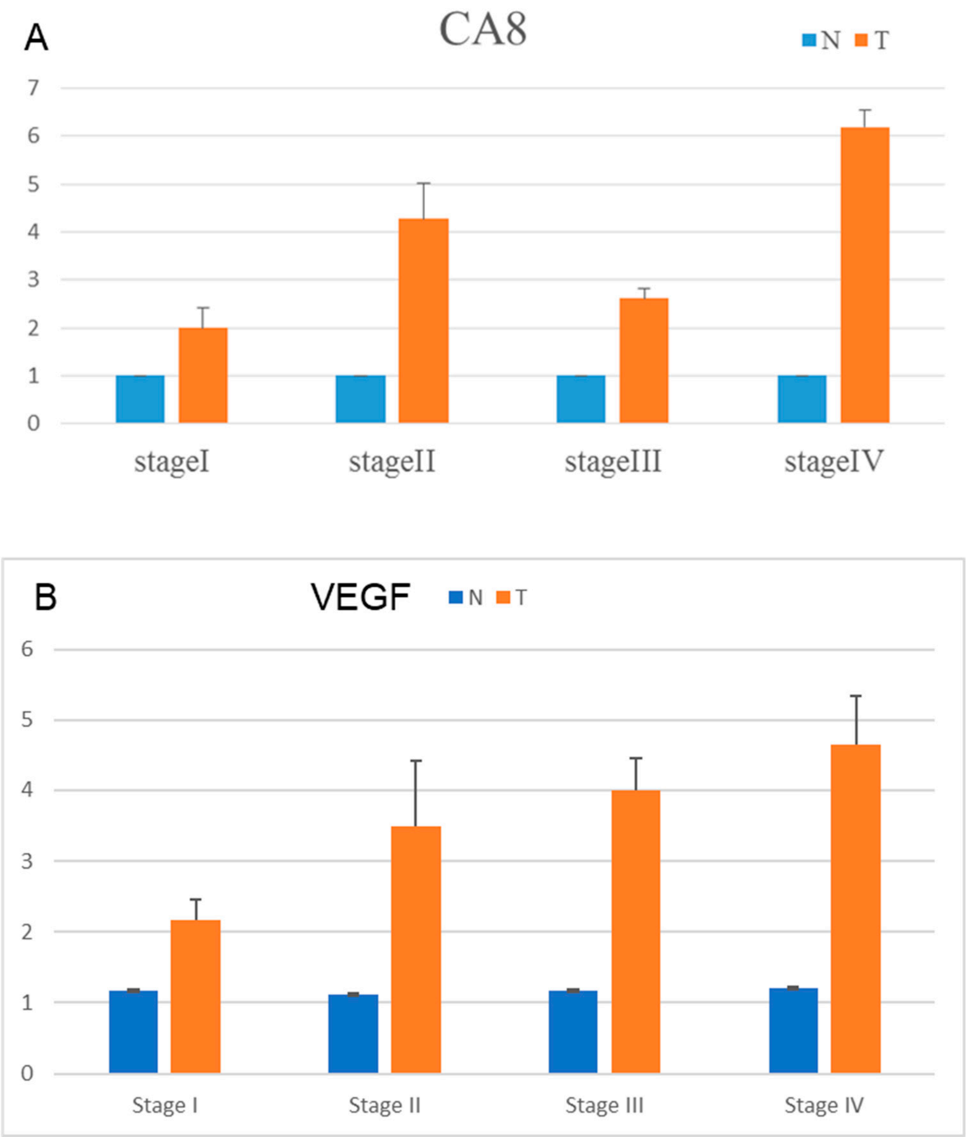

Figure S3. Expression of PC in paraffin-embedded tissues of CRC patients with stage I, II, III and IV by IHC staining. indicate the non-cancerous area adjacent to the cancerous area in CRC stage III and IV, respectively. Orange bar indicate cancer and blue bar indicate stromal area within tissues with CRC.
